# Combined Associations of Smoking and Bullying Victimization With Binge Drinking Among Adolescents in Beijing, China

**DOI:** 10.3389/fpsyt.2021.698562

**Published:** 2021-09-16

**Authors:** Li Chen, Ruo-Ran Lu, Jia-Li Duan, Jun Ma, Guangrong Zhu, Yi Song, Patrick W. C. Lau, Judith J. Prochaska

**Affiliations:** ^1^School of Public Health, Institute of Child and Adolescent Health, Peking University, Beijing, China; ^2^Beijing Center for Disease Prevention and Control, Beijing, China; ^3^Department of Sport, Physical Education and Health, Hong Kong Baptist University, Hong Kong, SAR China; ^4^Laboratory of Exercise Science and Health, BNU-HKBU United International College, Zhuhai, China; ^5^Department of Medicine, Stanford Prevention Research Center, Stanford University, Stanford, CA, United States

**Keywords:** individual associations, combined associations, binge drinking, smoking, bullying victimization, adolescents

## Abstract

**Background:** Binge drinking and smoking among adolescents are serious public concerns. However, very few studies have explored the reinforcement of bullying victimization by such behavior. Our study aimed at examining the individual and combined associations of smoking and bullying victimization with binge drinking among adolescents in Beijing, China.

**Methods:** A total of 33,694 students aged 13–17 years old in Beijing, China were anonymously investigated via the cross-sectional Chinese Youth Risk Behavior Surveillance Survey from April to May 2014. A three-stage stratified sampling was used to select participants. Factors such as sociodemographic variables and indicators of smoking, bullying victimization, and binge drinking were analyzed with multiple logistic regressions, and joint and additive interaction effects were tested.

**Results:** Overall, ever-drinking prevalence was 59.1% (boys: 64.4%; girls: 53.7%). Past 30-day binge drinking was 11.5% (boys: 15.6%; girls: 7.4%) and frequent binge drinking was 2.3% (boys: 3.3%; girls: 1.0%). Past 30-day smoking was 10.7% (boys: 16.4%; girls: 5.0%) and past 30-day bullying victimization was 48.7% (boys: 57.3%; girls: 40.1%). The combined effects of smoking and bullying victimization on occasional binge drinking (OR = 6.49, 95% CI = 5.60–7.52) and frequent binge drinking (OR = 10.32, 95% CI = 7.52–14.14) were significant, and the additive interaction effect was significant for current smoking and bullying victimization on frequent binge drinking (OR = 10.22, 95% CI = 9.43–11.07). The additive interaction effect for current smoking and bullying victimization on frequent binge drinking was significant among boys.

**Conclusion:** Bullying victimization reinforced the association of smoking with frequent binge drinking, especially with findings specific to boys. Programs to prevent smoking or bullying or both may reduce binge drinking among adolescents in China.

## Introduction

The use of alcohol significantly affects adolescents' physical and mental health around the world (1) and contributes to more than 200 diseases and injuries, causing about 25% of deaths in the 20–29 age group (2). As the world's largest producer and consumer of alcohol, China annually consumes an average of 6.7 liters per person, which is greater than the average of 6.2 liters worldwide (for those over 15 years old) (2). In China, adolescents' ever-drinking prevalence is reported to range from 50 to 66% (3, 4), and the prevalence of adolescent binge drinking—defined as drinking five or more alcoholic drinks within a 1-to-2-h period—ranges from 5 to 10% (5–7). Young people who binge drink are at a greater risk of developing alcohol dependence in adulthood (8, 9). Binge drinking among adolescents can change their neural structure and activity and increase the risk of developing an alcohol use disorder (10). However, neural damage caused by binge drinking during adolescence can be repaired after the drinking stops (11).

Previous research has identified that tobacco consumption and alcohol abuse are highly correlated with each other during adolescence (12), specifically with regards to smoking and binge drinking (13, 14). According to the China Youth Tobacco Survey, ~9.4 million middle school students have tried tobacco products and over 3 million are regular tobacco users (3). The prevalence of current drinking was 73.3% among current smokers and 16.6% among non-smokers. The prevalence of binge drinking (51.2%) was higher among current smokers than among non-smokers (6.8%) (15). Additionally, bullying victimization is also significantly associated with alcohol drinking and tobacco use during adolescence (16–19). Although the relationships between alcohol abuse, tobacco consumption, and bullying victimization in adolescents have been studied, there has only been limited research focused on the combined effects of tobacco use and bullying victimization on binge drinking, and it is less discussed in such interactions—especially in China. Although it is not difficult to find the co-occurrence of tobacco use, bullying victimization and binge drinking, sparse research considered the combined effects of tobacco use and bullying victimization on binge drinking. However, exploring these combined effects has important implications for developing adolescent health promotion strategies and reducing health inequalities among adolescents. Compared with the intervention targeting a single risk factor, the comprehensive intervention targeting multiple factors showed effectiveness for promoting adolescent health (20, 21).

We hypothesized that smoking and bullying victimization might influence binge drinking, whereby the effects would be additive. The combined effect of smoking and bullying victimization on binge drinking was larger than the sum of the individual effects. Bullying victimization might reinforce the association of smoking with binge drinking. The Chinese Youth Risk Behavior Survey (CYRBS) conducted in Beijing provided us an opportunity to assess the effects. In this large representative sample of Chinese adolescents, we examined overall binge drinking and frequent binge drinking—defined as binge drinking for more than 5 days in the past 30 days—and aimed to assess the strength of smoking and bullying victimization on binge drinking alone and as combined effects in interaction. Given the known differences in the prevalence of alcohol use according to gender, we separately tested the overall effects for boys and girls.

## Methods

### Study Design

A cross-sectional survey was conducted among 33,694 middle and high school students (aged 13–17) in Beijing, China, from April to May 2014. The sampling procedure, as previously described in (22, 23), was a three-stage stratified sampling method, which obtained a representative sample of students studying in grades 7–12. First, three districts or counties were sampled based on socioeconomic levels (upper, moderate, and lower) (23). Second, the middle and senior high schools were categorized as vocational (only for grade 10–12 students), ordinary, or key schools (24). The ordinary and key schools were categorized based on the teaching ability and skills of teachers and the average academic performance of the students (24). The students and teachers from key schools hold better academic performance and more teaching ability and skills. Based on probability proportional to school enrolment size, 31 vocational senior high schools (grade 10–12), 36 ordinary middle schools, 35 ordinary high schools, 27 key middle schools, and 36 key high schools were selected. Finally, a simple random sampling method was adopted to select n classes from each grade at each school (n depended on the average size of classes and was no <200 students per school). Parents' informed consent and students' assent were obtained. This study is based on the PPS sample (Probability-Proportional-to-Size Sampling) of schools, which can represent the students in Beijing. All respondents were informed that the survey would be anonymously conducted and that their privacy would be protected. Participants completed the self-administered questionnaire in their classroom without any supervision from teachers. The survey questionnaire we used was adapted from the 2003 YRBS in the United States with a high degree of reliability and validity (25). For our analysis, we included students from all sampling schools and classes and excluded participants with missing information and students younger than 12 or older than 18 years (response rate = 94.45%). Peking University's Medical Research Ethics Committee approved the study protocol (IRB00001052-17010).

### Sociodemographic Variables

After reviewing previous studies on binge drinking, we identified and adjusted for the following binge drinking-related factors in our study (26). Sociodemographic information included gender (boys and girls), age, school type (middle and high school), mother's education level (middle school, high school/technical school/technical secondary school/junior college, graduate of the university and above, and not sure), boarding students (no and yes), academic performance in the past 12 months, past 7-day television screen-time (none, <1, 1–3, and >3 h), and past 7-day video game-time (none, <1, 1–3, and >3 h) (27). Academic performance was assessed by the following self-reported question: “How would you describe your grades in class?” Answer options included “excellent,” “above-average,” “average,” “below-average,” “poor,” and “not sure,” and these were further categorized into the following four groups: poor (included: poor), average (included: above-average, average, and below-average), excellent (included: excellent), and not sure (included: not sure).

The feeling of loneliness was measured by “How often you feel lonely in the past 12 months,” and the answer options included “never,” “rarely or sometimes,” and “often or always.” The fighting experience was assessed by “How many times have you fought with others in the past 12 months,” and the answer options included none, 1–3 times, 4–5 times, and >5 times.

### Alcohol Consumption and Binge Drinking

Alcohol use was determined by a report of having consumed at least one drink of alcohol in one's lifetime. Never drinkers were defined as participants who never consumed alcohol. Participants who consumed alcohol were considered as ever drinkers if they reported not drinking alcohol during the past 30 days. Binge drinking was defined as drinking at least five glasses of alcohol within 1–2 h. Among ever drinkers, past 30-day binge drinking was classified as “none (0 days)” and “binge drinking (1–30 days)”; we also divided “binge drinking (1–30 days)” into “occasional binge drinking (1–5 days)” and “frequent binge drinking (6–30 days)” (28). Additionally, the age at which alcohol was first consumed, the reasons for drinking, frequency of drinking in the past 30 days, and the frequency of drunkenness symptoms in the past 12 months were reported. The choices listed for the reason of drinking were “drink with family/friends,” “coercion by others,” “curiosity,” “bad mood,” “happy,” “habit,” “unavailability of other drinks,” and “other reasons.”

### Smoking Status and Bullying Victimization

The smoking status was assessed by asking the question: “Have you ever tried cigarette smoking, even one or two puffs?” Participants who answered “No” were defined as never smokers and participants who answered “Yes” but not smoking in the past 30 days were coded as former smokers. Current smoking was defined as smoking a cigarette in the past 30 days (23).

Bullying victimization was measured by asking, During the past 30 days, (A) have you been maliciously teased? (B) have you been made fun of using gender-related jokes, comments, or gestures? (C) has anyone made fun of your individual physical imperfections or appearance? (D) have you been deliberately excluded from a group activity? (E) have you been threatened or intimidated? (F) have you been blackmailed for money? or (G) have you been hit, kicked, pushed, squeezed, or locked indoors? Different types of victimization experiences were presented as separate items. A report of “Yes” on any of the victimization experiences was categorized as positive for bullying victimization. First, we examined different types of victimization experiences and then a composite of any bullying experience.

### Data Analysis

**Figure 1:**
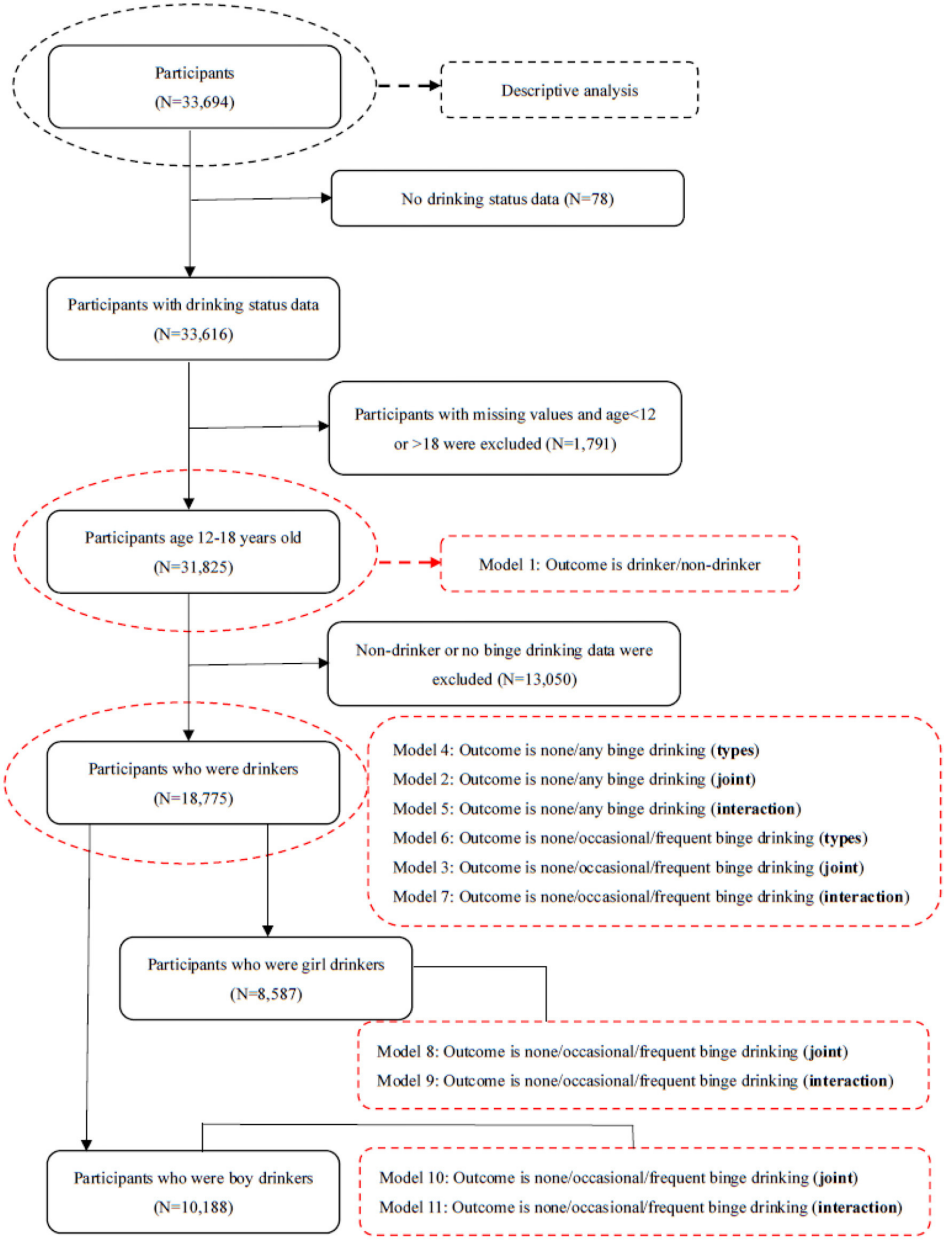
Data process description. Notes: types mean the different types of bullying victimization; joint means the joint effects; interaction means the interaction effects.

Frequencies were calculated to summarize the distributions of the categorical variables. Chi-square tests were performed to examine the distributions of the categorical behavioral variables with gender and school level (middle vs. high school). The binary logistic model (model 1) was developed using sociodemographic factors, smoking status (never, former, current), and different types of victimization experiences as independent variables and drinking status (drinkers/non-drinkers) as the dependent variable. Among drinkers, multinomial logistic models were established to explore the joint effect of smoking and bullying victimization on binge drinking (model 2 and model 3). To explore this interaction effect of smoking and bullying victimization on binge drinking, logistic models were established with bullying victimization × smoking as the interaction item. To explore the difference between the interaction effects of binge drinking on boys and girls, a gender stratification analysis was conducted. The presence and direction of interaction depend on the scale, e.g., additive or multiplicative. Additive interaction means that the combined effect of two exposures is larger (or smaller) than the sum of the individual effects, and multiplicative interaction means that the combined effect is larger (or smaller) than the product of the individual effects of the two exposures (29). All models above were controlled for sociodemographic factors. In the models above, the interaction effect of bullying victimization × smoking was tested. If the multiplicative interaction was not found to be significant, then we explored the additive interaction effect on binge drinking by using Andersson's Excel sheet (30). The data processing has been shown in Figure 1 and the information of models has been shown in Table 1. Statistical tests were two-sided, and statistical significance was set at *p* < 0.05. All data were analyzed using SPSS 22.0 for Windows (SPSS Inc., Chicago, IL, USA).

**Table 1:** Characteristics of sociodemographic variables overall and by gender, *N* = 33,694.

**Variables**	**Boys**	**Girls**	**Total**	**Pearson *X^**2**^***	***P*-value**
	***N* (%)**	***N* (%)**	***N* (%)**		
**School type**
Middle school	7,756 (46.0)	7,178 (42.7)	14,934 (44.3)	36.67	<0.001
High school	9,119 (54.0)	9,641 (57.3)	18,760 (55.7)		
**Mother‘s education level**
JHSB^a^	5,320 (31.8)	5,541 (33.1)	10,861 (32.5)	95.58	<0.001
STTJ^b^	6,731 (40.2)	7,177 (42.9)	13,908 (41.6)		
Graduate of university and above	3,566 (21.3)	3,253 (19.4)	6,819 (20.4)		
Not sure	1,108 (6.6)	766 (4.6)	1,874 (5.6)		
**Boarding students**
Yes	3,645 (21.9)	4,422 (26.5)	8,067 (24.2)	96.68	<0.001
No	13,025 (78.1)	12,281 (73.5)	25,306 (75.8)		
**School achievement**
Poor	1,762 (10.7)	914 (5.5)	2,676 (8.1)	375.17	<0.001
Middle	11,639 (70.7)	12,543 (75.7)	24,182 (73.2)		
Excellent	2,109 (12.8)	2,430 (14.7)	4,539 (13.7)		
Not sure	964 (5.9)	677 (4.1)	1,641 (5.0)		
**Frequency of fighting, past 12 months**
None	11,777 (69.9)	15,225 (90.6)	27,002 (80.2)	2297.17	<0.001
1–3 times	3,965 (23.5)	1,315 (7.8)	5,280 (15.7)		
4–5 times	482 (2.9)	117 (0.7)	599 (1.8)		
≥ 6 times	635 (3.8)	147 (0.9)	782 (2.3)		
**Feeling lonely**
Never	6,265 (37.2)	4,806 (28.6)	11,071 (32.9)	398.77	<0.001
Rarely or sometimes	8,407 (49.8)	9,721 (57.8)	18,128 (53.9)		
Often or always	2,190 (13.0)	2,279 (13.6)	4,469 (13.3)		
**Television screen-time**
None	3,088 (18.3)	2,544 (15.1)	5,632 (16.7)	83.24	<0.001
<1 h	5,735 (34.1)	6,336 (37.7)	12,071 (35.9)		
1–3 h	6,746 (40.1)	6,642 (39.5)	13,388 (39.8)		
≥ 4 h	1,271 (7.5)	1,277 (7.6)	2,548 (7.6)		
**Video game-time**
None	2,236 (13.5)	2,629 (15.7)	4,892 (14.6)	420.59	<0.001
<1 h	4,499 (26.8)	5,590 (33.4)	10,089 (30.1)		
1–3 h	6,939 (41.3)	6,600 (39.4)	13,539 (40.3)		
≥ 4 h	2,675 (15.9)	2,480 (14.8)	5,155 (15.4)		

a *JHSB, Middle school and below.*

b *STTJ, Senior high school/technical school/technical secondary school/junior college*.

## Results

### Participants

This sample of 33,694 students was largely balanced for gender (50.1% male, 49.9% female) and school level (44.3% middle school, 55.7% high school); 32.5% reported their mothers' highest education was middle school and below; 24.2% were boarding students, and 73.2% reported average academic achievement. Overall, 19.8% reported a record of fighting (30.1% of boys, 9.4% of girls), 7.6% watched television 4+ h per day, and 15.4% played video games 4+ h per day (Table 1).

### Alcohol Consumption and Binge Drinking

Overall, 59.1% reported ever drinking alcohol, higher among boys (64.4%) than girls (53.7%) and among high school students (67.5%) than middle school students (48.5%). Table 2 summarizes the variable of alcohol use by gender and school level. Among ever drinkers, drinking for the first time mostly occurred between the ages of 12 and 15. Boys started drinking earlier than girls. Among ever drinkers, the main reason for drinking alcohol was drinking with family/friends. Boys drank more with family or friends than girls, and high school students drank more with family/friends than middle school students.

**Table 2:** Characteristics of alcohol consumption and binge drinking by gender and school level, N (%).

**Variables**	**Boys**	**Girls**	***X^**2**^* test**	**Middle school**	**High school**	***X^**2**^* test**	**Total**
**Drinking status**							
Ever drinker	10,840 (64.4)	9,018 (53.7)		7,213 (48.5)	12,645 (67.5)		19,858 (59.1)
			*X*^2^ = 397.41 *P < * 0.001			*X*^2^ = 1243 *P < * 0.001	
Never drinker	5,989 (35.6)	7,769 (46.3)		7,670 (51.5)	6,088 (32.5)		13,758 (40.9)
**The age of first drinking, among ever drinkers**							
Never	818 (7.6)	847 (9.4)		907 (7.2)	758 (10.5)		1,665 (8.4)
			*X*^2^ = 154.31 *P < * 0.001			*X*^2^ = 2504.53 *P* <0.001	
≤ 7	2,175 (20.1)	1,497 (16.6)		2,291 (18.1)	1,381 (19.2)		3,672 (18.5)
8–9	998 (9.2)	591 (6.6)		820 (6.5)	769 (10.7)		1,589 (8.0)
10–11	1,254 (11.6)	882 (9.8)		894 (7.1)	1,242 (17.2)		2,136 (10.8)
12–13	2,138 (19.7)	1,863 (20.7)		2,007 (15.9)	1,994 (27.7)		4,001 (20.2)
14–15	2,243 (20.7)	2,078 (23.1)		3,303 (26.1)	1,018 (14.1)		4,321 (21.8)
≥16	1,205 (11.1)	1,249 (13.9)		2,415 (19.1)	39 (0.5)		2,454 (12.4)
**The reason for drinking among ever drinkers**							
Not drinking	1,536 (14.3)	1,491 (16.6)		1,650 (13.1)	1,377 (19.1)		3,027 (15.3)
			*X*^2^ = 188.29 *P* <0.001			*X*^2^ = 507.75 *P* <0.001	
Drink with family/friends	5,055 (46.9)	3,917 (43.6)		6,228 (49.6)	2,744 (38.1)		8,972 (45.4)
Others forced	351 (3.3)	189 (2.1)		341 (2.7)	199 (2.8)		540 (2.7)
Curiosity	544 (5.0)	485 (5.4)		455 (3.6)	574 (8.0)		1,029 (5.2)
Bad mood	1,017 (9.4)	1,174 (13.1)		1,470 (11.7)	721 (10.0)		2,191 (11.1)
Happy	1,102 (10.2)	661 (7.4)		1,175 (9.4)	588 (8.2)		1,763 (8.9)
Habit	269 (2.5)	169 (1.9)		269 (2.1)	169 (2.3)		438 (2.2)
No other drinks	301 (2.8)	247 (2.7)		264 (2.1)	284 (3.9)		548 (2.8)
Other reasons	602 (5.6)	654 (7.3)		712 (5.7)	544 (7.6)		1,256 (6.4)
**Frequency of drinking last 30 days, among ever drinkers**							
Not drinking	4,792 (44.3)	4,802 (53.4)		5,969 (47.3)	3,625 (50.5)		9,594 (48.4)
			*X*^2^ = 269.19 *P* <0.001			*X*^2^ = 44.57 *P* <0.001	
1–2 days	3,404 (31.5)	2,755 (30.6)		3,959 (31.4)	2,200 (30.6)		6,159 (31.1)
3–5 days	1,139 (10.5)	722 (8.0)		1,214 (9.6)	647 (9.0)		1,861 (9.4)
6–9 days	477 (4.4)	230 (2.6)		443 (3.5)	264 (3.7)		707 (3.6)
10–19 days	439 (4.1)	184 (2.0)		434 (3.4)	189 (2.6)		623 (3.1)
20–29 days	149 (1.4)	90 (1.0)		154 (1.2)	85 (1.2)		239 (1.2)
30 days	414 (3.8)	209 (2.3)		454 (3.6)	169 (2.4)		623 (3.1)
**Binge drinking last 30 days, among ever drinkers**							
None	8,306 (76.8)	7,796 (86.6)		9,953 (78.9)	6,149 (85.4)		16,102 (81.3)
			*X*^2^ = 345.27 *P* <0.001			*X*^2^ = 134.46 *P* <0.001	
1–2 days	1,535 (14.2)	866 (9.6)		1,680 (13.3)	721 (10.0)		2,401 (12.1)
3–5 days	465 (4.3)	188 (2.1)		490 (3.9)	163 (2.3)		653 (3.3)
6-9 days	215 (2.0)	76 (0.8)		212 (1.7)	79 (1.1)		291 (1.5)
10–19 days	116 (1.1)	30 (0.3)		112 (0.9)	34 (0.5)		146 (0.7)
20-29 days	34 (0.3)	15 (0.2)		35 (0.3)	14 (0.2)		49 (0.2)
30 days	139 (1.3)	35 (0.4)		131 (1.0)	43 (0.6)		174 (0.9)
**Frequency of drunkenness symptoms last 12 months, among ever drinkers**							
None	8,307 (77.0)	7,442 (83.0)		9,626 (76.6)	6,123 (85.0)		15,749 (79.7)
			*X*^2^ = 203.26 *P* <0.001			*X*^2^ = 598.18 *P* <0.001	
1–2 times	1,885 (17.5)	1,342 (15.0)		2,317 (18.4)	910 (12.6)		3,227 (16.3)
3–9 times	408 (3.8)	151 (1.7)		433 (3.4)	126 (1.7)		559 (2.8)
More than 9 times	195 (1.8)	34 (0.4)		183 (1.5)	46 (0.6)		229 (1.2)

For the full sample, past 30-day alcohol use was reported by 31.5% of students, higher among boys (37.2%) than girls (25.8%) and among high school students (36.8%) than middle school students (24.8%). Among ever drinkers, past 30-day alcohol use was reported by 51.6% of students (55.7% of boys and 46.6% of girls). For the full sample, past 30-day binge drinking was reported by 11.5% of students, higher among boys (15.6%) than girls (7.4%) and among high school students (14.8%) than middle school students (7.3%). Among ever drinkers, past 30-day binge drinking was reported by 18.7% of students (23.2% of boys and 13.4% of girls). Among ever drinkers, symptoms of drunkenness were reported by 20.3% of students (Table 2).

### Smoking and Bullying Victimization

Overall, 24.3% reported former smoking (31.8% of boys and 16.8% of girls), and 10.7% reported past 30-day smoking (16.4% of boys and 5.0% of girls). More high school students (31.2%) than middle school students (15.5%) reported former smoking and more high school students (14.9%) than middle school students (5.4%) reported current smoking.

By frequency, the top four bullying victimization behaviors were being maliciously teased (35.9%); being made fun of by gender-related jokes, comments, or gestures (21.7%); being made fun of individual physical appearance (15.5%), and being deliberately excluded from a group activity (13.1%). Across all categories of bullying, except for being made fun of physical defect or appearance, boys were more likely to be bullied than girls (Table 3).

**Table 3:** Characteristics of smoking and bullying victimization by gender and school level.

**Variables**		**Boys**	**Girls**	***X*^**2**^ test**	**Middle school**	**High school**	***X*^**2**^ test**	**Total**
		***N* (%)**	***N* (%)**		***N* (%)**	***N* (%)**		***N* (%)**
**Smoking status**								
Never smoker		11,290 (68.2)	13,786 (83.2)		12,249 (84.5)	12,827 (68.8)		25,076 (75.7)
				*X*^2^ = 1324.33 *P* <0.001			*X*^2^ = 1188.53 *P* <0.001	
Former smoker		2,548 (15.4)	1,948 (11.8)		1,458 (10.1)	3,038 (16.3)		4,496 (13.6)
Current smoker		2,717 (16.4)	836 (5.0)		778 (5.4)	2,775 (14.9)		3,553 (10.7)
**Different type bullying victimization**								
Bullying (A)	NO	9,440 (56.0)	12,130 (72.2)		8,979 (60.2)	12,591 (67.2)		21,570 (64.1)
				*X*^2^ = 959.88 *P* <0.001			*X*^2^ = 177.98 P <0.001	
	YES	7,420 (44.0)	4,672 (27.8)		5,944 (39.8)	6,148 (32.8)		12,092 (35.9)
Bullying (B)	NO	11,732 (69.6)	14,634 (87.1)		11,946 (80.1)	14,420 (76.9)		26,366 (78.3)
				*X*^2^ = 1515.99 *P* <0.001			*X*^2^ = 49.74 *P* <0.001	
	YES	5,126 (30.4)	2,171 (12.9)		2,968 (19.9)	4,329 (23.1)		7,297 (21.7)
Bullying (C)	NO	14,197 (84.2)	14,261 (84.8)		12,738 (85.4)	15,720 (83.8)		28,458 (84.5)
				*X*^2^ = 2.61 *P* = 0.107			*X*^2^ = 16.72 *P* <0.001	
	YES	2,663 (15.8)	2,548 (15.2)		2,173 (14.6)	3,038 (16.2)		5,211 (15.5)
Bullying (D)	NO	14,396 (85.4)	14,861 (88.4)		12,774 (85.6)	16,483 (87.9)		29,257 (86.9)
				*X*^2^=67.47 *P* <0.001			1*X*^2^ = 37.60 *P* <0.001	
	YES	2,462 (14.6)	1,947 (11.6)		2,142 (14.4)	2,267 (12.2)		4,409 (13.1)
Bullying (E)	NO	15,281 (90.6)	16,027 (95.3)		13,721 (91.9)	17,587 (93.8)		31,308 (93.0)
				*X*^2^ = 290.66 *P* <0.001			*X*^2^ = 42.90 *P* <0.001	
	YES	1,586 (9.4)	782 (4.7)		1,202 (8.1)	1,166 (6.2)		2,368 (7.0)
Bullying (F)	NO	15,452 (91.6)	16,152 (96.1)		13,865 (92.9)	17,739 (94.6)		31,604 (93.9)
				*X*^2^ = 293.57 *P* <0.001			*X*^2^ = 42.11 *P* <0.001	
	YES	1,415 (8.4)	656 (3.9)		1,060 (7.1)	1,011 (5.4)		2,071 (6.1)
Bullying (G)	NO	15,578 (92.4)	16,298 (97.0)		13,984 (93.8)	17,892 (95.4)		31,876 (94.7)
				*X*^2^ = 351.98 *P* <0.001			*X*^2^ = 43.60 *P* <0.001	
	YES	1,279 (7.6)	505 (3.0)		925 (6.2)	859 (4.6)		1,784 (5.3)
**Any Bullying victimization**								
NO	7,168 (42.7)	10,039 (59.9)		7,165 (48.2)	10,042 (53.7)		17,207 (51.3)	
				*X*^2^ = 998.51 *P* <0.001			*X*^2^ = 99.47 *P* <0.001	
YES	9,623 (57.3)	6,710 (40.1)		7,685 (51.8)	8,648 (46.3)		16,333 (48.7)	

*(A) Maliciously teased; (B) Made fun of with gender jokes, comments or gestures; (C) Been made fun of due to individual physical imperfections or looks; (D) Deliberately excluded from a collective activity; (E) Threatened or intimidated; (F) Demanded to give money; (G) Been hit, kicked, pushed, squeezed, or locked indoors*.

### Associations of Smoking and Bullying Victimization With Ever Drinking

Compared with never smokers, former smokers (OR = 4.28, 95% CI = 3.91–4.68) and current smokers (OR = 4.63, 95% CI = 4.13–5.20) were more likely to report ever drinking. The bullying victimization behaviors of being (A) maliciously teased (OR = 1.11, 95% CI = 1.05–1.18); (B) made fun of gender jokes, comments, or gestures (OR = 1.60, 95% CI = 1.49–1.72); and (C) made fun of individual physical imperfections or appearance (OR = 1.12, 95% CI = 1.03–1.21) were associated with ever drinking; while (D) being deliberately excluded from a group activity (OR = 0.81, 95% CI = 0.74–0.88) and (G) being hit, kicked, pushed, squeezed or locked in a room (OR = 0.73, 95% CI = 0.64–0.84) were associated with less likelihood of ever drinking. The bullying victimization behaviors of being (E) threatened or intimidated and (F) blackmailed for money were not significantly associated with ever drinking (Table 4). In addition to gender and school type, age, boarding at school, mother's education level, and academic performance were significantly associated with ever drinking (Supplementary Table 13).

**Table 4:** Multivariate logistic model for factors associated with ever drinking, *N* = 31,825 (model 1).

**Variables**		**Outcome is drinker/non-drinker**	
		**OR**	**95% CI**
Smoking	Never (ref)	1.00	
	Former	4.28	3.91–4.68
	Current	4.63	4.13–5.20
Bullying (A)	No (ref)	1.00	
	Yes	1.11	1.05–1.18
Bullying (B)	No (ref)	1.00	
	Yes	1.60	1.49–1.72
Bullying (C)	No (ref)	1.00	
	Yes	1.12	1.03–1.21
Bullying (D)	No (ref)	1.00	
	Yes	0.81	0.74–0.88
Bullying (E)	No (ref)	1.00	
	Yes	0.92	0.81–1.04
Bullying (F)	No (ref)	1.00	
	Yes	1.01	0.89–1.14
Bullying (G)	No (ref)	1.00	
	Yes	0.73	0.64–0.84

*(A) Maliciously teased (B) Made fun of with gender jokes, comments or gestures (C) Been made fun of due to individual physical imperfections or looks (D) Deliberately excluded from or outside of a collective activity (E) Threatened or intimidated (F) Blackmailed or forced to give money (G) Been hit, kicked, pushed, squeezed, or locked indoors. Model 1: The logistic regression model for smoking and different type of bullying victimization affecting drinking status adjusting for sociodemographic factors among all participants*.

### Smoking and Bullying Victimization Interacted in Their Association With Binge Drinking, Among Ever Drinkers

The remaining analysis was restricted to ever drinkers. Compared with never smokers who were not bullied, students who were former smokers and victims of bullying (OR = 1.91, 95% CI = 1.66–2.21), and students who were current smokers and victims of bullying (OR = 7.01, 95% CI = 6.10–8.05) were prone to a greater risk of binge drinking. The findings were identical to those of occasional binge drinking (Table 5 and Supplementary Table 14). The additive interaction effects of former/current smoking and bullying victimization experience on any binge drinking (1–30 days) were not significant, while the additive effects on occasional binge drinking were also not significant (Supplementary Tables 1–6).

**Table 5:** Multinomial logistic model for joint effects of smoking and bullying victimization on binge drinking among ever drinkers by adjustment for sociodemographic factors.

**Variables**	**Model 2**, ***N*****=18,775**		**Model 3**, ***N*****=18,775**			
	**Outcome is none/binge drinking (1–30 days)** ^**a**^		**Occasional binge drinking (1–5 days)** ^**b**^		**Frequent binge drinking (6–30 days)** ^**c**^	
	**OR**	**95% CI**	**OR**	**95% CI**	**OR**	**95% CI**
**Joint effects of smoking and bullying victimization**						
*Never smoker × No victimization experience*	1.00		1.00		1.00	
*Never smoker × Yes victimization experiences*	1.30	1.15–1.47	1.29	1.13–1.47	1.44	1.05–1.99
*Former smoker × No victimization experience*	1.82	1.54–2.16	1.89	1.59–2.26	1.41	0.88–2.27
*Former smoker × Yes victimization experiences*	1.91	1.66–2.21	1.96	1.68–2.28	1.76	1.21–2.55
*Current smoker × No victimization experience*	5.93	5.06–6.96	5.53	4.66–6.55	8.57	6.08–12.08
*Current smoker × Yes victimization experiences*	7.01	6.10–8.05	6.49	5.60–7.52	10.32	7.52–14.14

a, b, c *the reference is none (never binge drinking)*.

Some sociodemographic variables were significantly associated with binge drinking (1–30 days) among ever drinkers (Supplementary Table 14).

**Figure 2:**
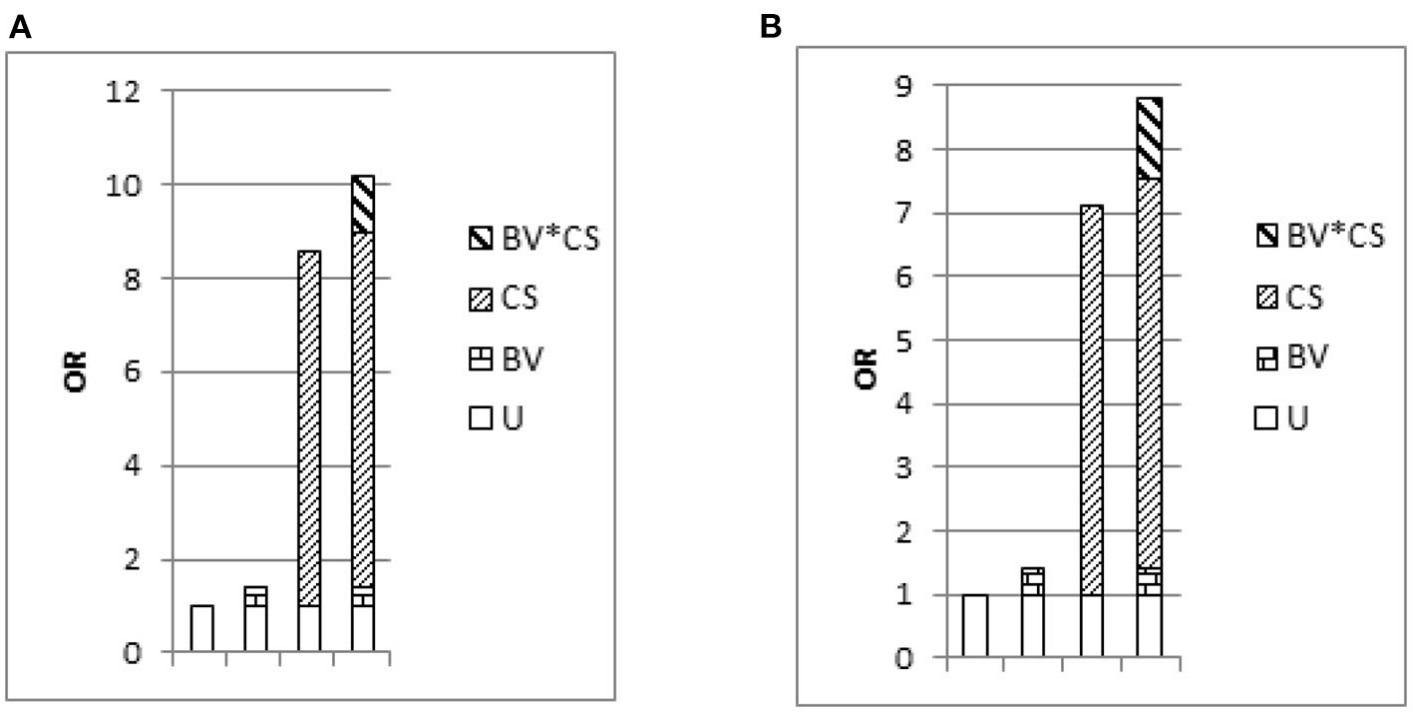
Odds ratio with contributions from bullying victimization and current smoking marked. Notes: U is the common reference category (OR = 1); BV is bullying victimization; CS is current smoking; **(A)** outcome is none/frequent binge drinking, among drinkers, *N* = 15,109; **(B)** outcome is none/frequent binge drinking, among boy drinkers, *N* = 8,119.

Students who were found to be binge drinking had a history of smoking and experience of bullying in their lives (Table 5). Moreover, a significant additive interaction effect for current smoking and bullying victimization on frequent binge drinking (OR = 10.22, 95% CI = 9.43–11.07) was observed (Figure 2A and Supplementary Table 6).

When stratified according to gender, the findings were generally similar (Supplementary Tables 7–12); however, the statistically significant associations between binge drinking with academic performance and feeling lonely were observed only among boys (Supplementary Tables 7, 10). Further, a significant additive interaction effect was found between current smoking and bullying victimization on frequent binge drinking among boys but not girls (Figure 2B and Supplementary Tables 7–9).

## Discussion

In this large scale adolescent age sample, the ever-drinking prevalence was 59.1%, which was consistent with other reports on adolescents' ever drinking prevalence in China, which ranged from 50 to 67% (3, 4, 31). In comparison to this, adolescent ever drinking was globally reported at 37.5%, 46.5% in the Western Pacific Region (which includes China), and at 70.2% for the Region of the Americas (70.2%), the latter being the highest across the world (32). The binge drinking prevalence was 11.5% in the current sample, which was higher than previous studies conducted in China (6, 15), yet lower than some western countries like the United States (17.7%) and France (13.8%) (14, 33). The higher prevalence of binge drinking might be explained by the differences in geography and economics. The current study was conducted in Beijing, the capital of China, which lies in the northern China, where the culture of drinking is widespread, and Beijing‘s economic level is higher than those cities where previous studies were conducted.

In the global status report on alcohol and health 2018, the WHO suggested that the Western Pacific Region should raise awareness and advocacy of alcohol-related harm (32). The action program on healthy lifestyles issued by the Beijing Municipal Government pointed out that children and adolescents were the target audience for raising awareness about the harm caused by drinking (34). The WHO also recommended that raising the legal age for buying alcohol would be an effective way to prevent young people from drinking (32). China prohibits the sale of alcohol to persons under 18. Raising the legal drinking age further to age 21 could significantly reduce adolescent drinking (35). In the current study, adolescents reported drinking alcohol mostly with family and friends. In traditional Chinese culture, drinking alcohol is considered a type of socializing, especially in business practices (36). Our finding supports that the adolescent's drinking behavior was tolerated by his or her family (37). The programs designed to prevent teen drinking mentioned above are not well-implemented, which is partly attributed to the fact that alcohol use is not perceived as a severe problem by most of the parents, and thus the regulation of “not drinking” under 18 has not been strictly implemented in China. As a consequence, the adolescents tend to mimic their parents' drinking behavior and share this experience with their peers (38). Health education expands not only to the adolescents but to their parents.

In the present study, former and current smoking were associated with binge drinking, which was consistent with previous studies (13, 14). We found that previously being a victim of bullying (a composite of the different types of bullying) was associated with binge drinking. Previous studies on the relationship between bullying victimization and binge drinking have produced mixed findings. For example, two studies in the United States found no significant relationship between traditional bullying victimization and binge drinking (39, 40); whereas, other studies showed that bullying victimization places one at higher risk for binge drinking (16, 41–43).

Our findings identified that bullying victimization reinforced the association of smoking with frequent binge drinking among boy drinkers, which indicates that increased attention should be paid to boys to prevent adolescent bullying and smoking and the efforts might be more beneficial for boys than girls. However, concerning the effects of interaction between smoking and the experience of any bullying victimization on occasional binge drinking, we did not find significant interaction effects of smoking and the experience of any bullying victimization on occasional binge drinking, which meant that the former smoking and experience of any bullying victimization individually influenced the occasional binge drinking instead of as a combined effect. To our knowledge, this study is the first to report on the interaction between smoking and bullying victimization on frequent binge drinking in China. The additive interactive effect on frequent binge drinking was significantly higher than the sum of the individual effects. The findings support efforts by governmental health and education agencies, local schools, and parents to prevent adolescent smoking and bullying, which might help prevent binge drinking. An example of such efforts was China's Ministry of Education's special governance on school bullying prevention that was released in 2016, to reduce the occurrence of school bullying (44).

For the intervention on binge drinking, the problem behavior theory might be an effective theory (45), which has been applied for many risk behavior interventions (46, 47). Our findings suggest that future intervention should consider the combined effects of different risk behaviors. Previous study focused on suicide prevention showed that school-based prevention programs might be the effective way to prevent adolescent risk behaviors (48). This evidence suggests that future intervention for adolescents need a comprehensive, school-based intervention targeting multiple risk behaviors.

A positive association was found with ever drinking (being maliciously teased; made fun of with gender jokes, comments, or gestures; and being made fun of based on individual physical appearance) in three of the seven bullying victimization experiences that we examined; whereas, two of the bullying victimization behaviors (being deliberately excluded from a group activity and being hit, kicked, pushed, squeezed, or locked indoors) were associated with a lower likelihood of ever drinking. The three bullying victimization experiences that were positively related were all verbal bullying. Physical bullying was directly reflected in physical injuries, and teachers and parents tended to conduct, in a timely fashion, psychological counseling for bullied students. However, verbal bullying was reflected psychologically, not physically, and was easily ignored by parents and teachers. Therefore, parents and teachers should not only focus on physical bullying, but also pay attention to students who are verbally bullied, and teach students how to properly protect themselves when they are bullied.

The present study covered a relatively large sample size and identified the effects of additive interaction between current smoking and bullying victimization on frequent binge drinking in adolescents in China. This study found the interaction between smoking, binge drinking, and bullying among Chinese adolescents, thereby providing theoretical support for the understanding of bullying and binge drinking among adolescents. It also provided theoretical support for bullying and binge drinking interventions in various regions. Limitations of this study included the cross-sectional design and the fact that all data were self-reported by students, which might be susceptible to recall and social desirability biases. Considering the self-reported variables, adolescents tend to underreport substance use and bullying victimization. Given the study's cross-sectional design, causation cannot be inferred from the associations identified. While bullying victimization and smoking might cause binge drinking, binge drinking might lead to current smoking and bullying victimization, or a third variable might drive the observed associations. For example, in adolescence, bullying victimization (49), alcohol intake (50), and smoking (51, 52) are all related to negative feelings. Given cultural differences, the additive interaction effects found in the present study warrant further study in other counties to see if the findings may generalize. While the bullying victimization involved in this study was traditional bullying victimization (not cyberbullying victimization), previous research has found that cyberbullying is also one of the main forms of bullying victimization (39, 40). The relationship between cyberbullying and alcoholism still needs further research and investigation. There are some possibilities of various confounders. For example, Father‘s education and occupation that influence binge drinking was not surveyed. However, previous studies showed that mother‘s education has a stronger effect on children‘s health behavior in societies where mothers are the main caregivers of the child (53).

## Conclusion

The prevalence of drinking and binge drinking among adolescents was 59.1 and 11.5%, respectively, in Beijing, China. It was higher among boys and high school students. Smoking and bullying victimization were significantly associated with binge drinking. Adjusting for sociodemographic factors, the significant additive interactive effects of smoking and bullying victimization on frequent binge drinking were observed, and they indicated that bullying victimization reinforces the effect of smoking on frequent binge drinking among boys who drink. Preventing smoking and bullying may help prevent binge drinking.

## Data Availability Statement

The original contributions presented in the study are included in the article/Supplementary Material, further inquiries can be directed to the corresponding author/s.

## Ethics Statement

The studies involving human participants were reviewed and approved by Peking University's Medical Research Ethics Committee approved the study protocol (IRB00001052-17010). Written informed consent to participate in this study was provided by the participants' legal guardian/next of kin.

## Author Contributions

LC and YS conceived and designed the study. LC carried out the initial analyses and prepared the first draft of the manuscript. YS, JP, JM, PL, and GZ critically reviewed and revised the manuscript. YS, J-LD, and R-RL conducted the research and collected the data. All authors read and approved the final manuscript.

## Funding

The present study was supported by the National Natural Science Foundation (Grant No. 81673192 to JM) and Humanities and Social Sciences Planning Fund Project, Ministry of Education, People's Republic of China (19YJA890022 to YS).

## Conflict of Interest

JP has provided consultation to pharmaceutical and technology companies that make medications and other treatments for quitting smoking and has served as an expert witness in lawsuits against the tobacco companies. The remaining authors declare that the research was conducted in the absence of any commercial or financial relationships that could be construed as a potential conflict of interest.

## Publisher's Note

All claims expressed in this article are solely those of the authors and do not necessarily represent those of their affiliated organizations, or those of the publisher, the editors and the reviewers. Any product that may be evaluated in this article, or claim that may be made by its manufacturer, is not guaranteed or endorsed by the publisher.

## Supplementary Material

Supplementary Table 1 The association between binge drinking, different types of bullying victimization, and socio-demographic characteristics as well as relevant health risk behaviors among ever drinkers (*N* = 18,775, model 4).

Supplementary Table 2 Additive effects of never/former smoking and bullying victimization on binge drinking (*N* = 15,801, model 5).

Supplementary Table 3 Additive effects of never/current smoking and bullying victimization on binge drinking (*N* = 15,109, model 5).

Supplementary Table 4 The association between occasional binge drinking/ frequent binge drinking, different types of bullying victimization, and socio-demographic characteristic as well as relevant health risk behaviors among ever drinkers (*N* = 18,775, model 6).

Supplementary Table 5 Additive effects of never/former smoking and bullying victimization on occasional binge drinking and frequent binge drinking (*N* = 15,801, model 7).

Supplementary Table 6 Additive effects of never/current smoking and bullying victimization on occasional binge drinking and frequent binge drinking (*N* = 15,109, model 7).

Supplementary Table 7 Multinomial logistic model for factors associated with occasional binge drinking and frequent binge drinking among boys, *N* = 10,188 (model 10).

Supplementary Table 8 Additive effects of never/former smoking and bullying victimization on occasional binge drinking and frequent binge drinking among boys (*N* = 7,915, model 11).

Supplementary Table 9 Additive effects of never/current smoking and bullying victimization on occasional binge drinking and frequent binge drinking among boys (*N* = 8,119, model 11).

Supplementary Table 10 Multinomial logistic model for factors associated with occasional binge drinking and frequent binge drinking among girls, *N* = 8,587 (model 8).

Supplementary Table 11 Additive effects of never/former smoking and bullying victimization on occasional binge drinking and frequent binge drinking among girls (*N* = 7,886, model 9).

Supplementary Table 12 Additive effects of never/current smoking and bullying victimization on occasional binge drinking and frequent binge drinking among girls (*N* = 6,990, model 9).

Supplementary Table 13 Multivariate logistic model for factors associated with ever drinking, *N* = 31,825 (model 1, full results).

Supplementary Table 14 Multinomial logistic model for smoking and bullying victimization interacted in their association with binge drinking among ever drinkers by adjustment for sociodemographic factors (full results).
