# Supplementary material for: Combined Associations of Smoking and Bullying Victimization With Binge Drinking Among Adolescents in Beijing, China
Source: Front Psychiatry. 2021 Sep 16;12:698562. doi: 10.3389/fpsyt.2021.698562 (PMC8481949; doi:10.3389/fpsyt.2021.698562)
Supplement: Supplementary Table 1 — The association between binge drinking, different types of bullying victimization, and socio-demographic characteristics as well as relevant health risk behaviors among ever drinkers (N = 18,775, model 4). [file Table_1.DOCX]

Supplementary Material

| Variables | | Outcome is none/any binge drinking (1-30 days) | |
| --- | --- | --- | --- |
|  |  | OR | 95% CI |
| Gender | Boys (ref) | 1.00 |  |
|  | Girls | 0.85 | 0.78 - 0.93 |
| Age |  | 1.05 | 1.00 - 1.10 |
| School type | Middle school | 1.00 |  |
|  | High school | 1.30 | 1.12 - 1.51 |
| Mother’s education level | JHSB | 1.00 |  |
|  | STTJ | 1.17 | 1.06 - 1.28 |
|  | Graduate and above | 1.18 | 1.04 - 1.33 |
|  | Not sure | 1.08 | 0.90 - 1.30 |
| Boarding students | Yes(ref) | 1.00 |  |
|  | No | 1.09 | 0.99 - 1.20 |
| School achievement | Poor | 1.00 |  |
|  | Middle | 0.98 | 0.88 - 1.09 |
|  | Excellent | 0.90 | 0.81 - 1.00 |
|  | Not sure | 1.15 | 0.98 - 1.35 |
| Fighting | 0 time | 1.00 |  |
|  | 1-3 times | 1.75 | 1.59 - 1.94 |
|  | 4-5 times | 2.84 | 2.27 - 3.55 |
|  | ≥6 times | 2.65 | 2.17 - 3.22 |
| Feeling lonely | Never | 1.00 |  |
|  | Rarely and sometimes | 1.06 | 0.96 - 1.18 |
|  | Often and always | 1.33 | 1.17 - 1.52 |
| Television screen-time | 0 hour | 1.00 |  |
|  | < 1 hour | 0.93 | 0.82 - 1.06 |
|  | 1-3 hours | 1.01 | 0.89 - 1.14 |
|  | ≥ 4 hours | 1.44 | 1.22 - 1.69 |
| Video game-time | 0 hour | 1.00 |  |
|  | < 1 hour | 0.97 | 0.82 - 1.14 |
|  | 1-3 hours | 1.29 | 1.11 - 1.50 |
|  | ≥ 4 hours | 1.65 | 1.41 - 1.94 |
| Smoking | Never smoking | 1.00 |  |
|  | Former smoking | 1.57 | 1.41 - 1.74 |
|  | Current smoking | 5.50 | 4.97 - 6.10 |
| Bullying (A) | No(ref) | 1.00 |  |
|  | Yes | 1.00 | 0.91 - 1.10 |
| Bullying (B) | No(ref) | 1.00 |  |
|  | Yes | 1.37 | 1.24 - 1.50 |
| Bullying (C) | No(ref) | 1.00 |  |
|  | Yes | 0.86 | 0.77 - 0.97 |
| Bullying (D) | No(ref) | 1.00 |  |
|  | Yes | 1.08 | 0.95 - 1.22 |
| Bullying (E) | No(ref) | 1.00 |  |
|  | Yes | 1.10 | 0.93 - 1.31 |
| Bullying (F) | No(ref) | 1.00 |  |
|  | Yes | 1.15 | 0.98 - 1.36 |
| Bullying (G) | No(ref) | 1.00 |  |
|  | Yes | 1.06 | 0.88 - 1.26 |

Table S2. Additive effects of **never/former smoking** and bullying victimization on **binge drinking** (N=15,801, model 5)

| **Jointed effects of never/former smoking and bullying victimization on any binge drinking** | | | | | | | |
| --- | --- | --- | --- | --- | --- | --- | --- |
| Bullying victimization | Smoking | | Bullying victimization & Smoking | | | β | OR（95%CI） |
| No | Never smoking | | 0 | | | — | 1.000 |
| Yes | Never smoking | | 1 | | | 0.247972 | 1.281(1.133 – 1.450) |
| No | Former smoking | | 2 | | | 0.595922 | 1.815(1.532 – 2.149) |
| Yes | Former smoking | | 3 | | | 0.631196 | 1.880(1.623 – 2.177) |
| **Variance and covariance matrix for never/ former smoking and bullying victimization** | | | | | | | |
| Items | | Bullying victimization | | Smoking | Bullying victimization & Smoking | | |
| Bullying victimization | | 0.003956 | | 0.002209 | 0.002485 | | |
| Smoking | | 0.002209 | | 0.007454 | 0.002364 | | |
| Bullying victimization & Smoking | | 0.002485 | | 0.002364 | 0.005610 | | |
| **Results of additive effects for never/former smoking and bullying victimization on any binge drinking using Andersson’s Excel** | | | | | | | |
| **Exposure** |  | |  | | | OR | 95 CI% |
| Bullying victimization | | | | | | 1.281 | 1.133 – 1.450 |
| Smoking | | | | | | 1.815 | 1.532 – 2.149 |
| Bullying victimization &Smoking | | | | | | 1.880 | 1.623 – 2.177 |
| **Measure** |  | |  | | | Estimate | 95 CI% |
| RERI |  | |  | | | -0.216 | -0.573 – 0.140 |
| AP |  | |  | | | -0.115 | -0.309 – 0.079 |
| S |  | |  | | | 0.803 | 0.569 – 1.133 |

Notes: RERI, the relative excess risk due to interaction; AP, the attributable proportion due to interaction; and S, the synergy index.

Table S3. Additive effects of **never/current smoking** and bullying victimization on **binge drinking** (N=15,109, model 5)

| **Jointed effects of never/** **current smoking and bullying victimization on any binge drinking** | | | | | | | |
| --- | --- | --- | --- | --- | --- | --- | --- |
| Bullying victimization | Smoking | | Bullying victimization & Smoking | | | β | OR（95%CI） |
| No | Never smoking | | 0 | | | — | 1.000 |
| Yes | Never smoking | | 1 | | | 0.237050 | 1.268(1.121 – 1.434) |
| No | Current smoking | | 2 | | | 1.744986 | 5.726(4.872 – 6.729) |
| Yes | Current smoking | | 3 | | | 1.900584 | 6.690(5.805 – 7.710) |
| **Variance and covariance matrix for never/current smoking and bullying victimization** | | | | | | | |
| Items | | Bullying victimization | | Smoking | Bullying victimization & Smoking | | |
| Bullying victimization | | 0.003949 | | 0.002287 | 0.002466 | | |
| Smoking | | 0.002287 | | 0.006781 | 0.002765 | | |
| Bullying victimization & Smoking | | 0.002466 | | 0.002765 | 0.005245 | | |
| **Results of additive effects for never/current smoking and bullying victimization on any binge drinking using Andersson’s Excel** | | | | | | | |
| **Exposure** |  | |  | | | OR | 95 CI% |
| Bullying victimization | | | | | | 1.268 | 1.121 – 1.434 |
| Smoking | | | | | | 5.726 | 4.872 – 6.729 |
| Bullying victimization &Smoking | | | | | | 6.690 | 5.805 – 7.710 |
| **Measure** |  | |  | | | Estimate | 95 CI% |
| RERI |  | |  | | | 0.696 | -0.270 – 1.663 |
| AP |  | |  | | | 0.104 | -0.034 – 0.242 |
| S |  | |  | | | 1.139 | 0.948 – 1.370 |

Notes: RERI, the relative excess risk due to interaction; AP, the attributable proportion due to interaction; and S, the synergy index.

Table S4. The association between occasional binge drinking/ frequent binge drinking, different types of bullying victimization and socio-demographic characteristic as well as relevant health risk behaviors among ever drinkers (N=18,775, model 6).

| Variables | | Occasional binge drinking (1-5 days) | | Frequent binge drinking (6-30 days) | |
| --- | --- | --- | --- | --- | --- |
|  |  | OR | 95% CI | OR | 95% CI |
| Gender | Boys (ref) | 1.00 |  | 1.00 |  |
|  | Girls | 0.89 | 0.81 - 0.98 | 0.66 | 0.54 - 0.82 |
| Age |  | 1.03 | 0.98 - 1.08 | 1.16 | 1.05 - 1.27 |
| School type | Middle school | 1.00 |  | 1.00 |  |
|  | High school | 1.30 | 1.11 - 1.53 | 1.28 | 0.92 - 1.77 |
| Mother’s education level | JHSB | 1.00 |  | 1.00 |  |
|  | STTJ | 1.17 | 1.06 - 1.30 | 1.13 | 0.92 - 1.39 |
|  | Graduate and above | 1.16 | 1.01 - 1.32 | 1.26 | 0.97 - 1.65 |
|  | Not sure | 1.04 | 0.85 - 1.26 | 1.27 | 0.90 - 1.78 |
| Boarding students | Yes(ref) | 1.00 |  | 1.00 |  |
|  | No | 1.06 | 0.96 - 1.17 | 1.22 | 0.99 - 1.50 |
| School achievement | Poor | 1.00 |  | 1.00 |  |
|  | Middle | 0.98 | 0.88 - 1.09 | 0.99 | 0.79 - 1.25 |
|  | Excellent | 0.84 | 0.76 - 0.94 | 1.24 | 0.99 - 1.54 |
|  | Not sure | 1.09 | 0.92 - 1.29 | 1.51 | 1.11 - 2.04 |
| Fighting | 0 time | 1.00 |  | 1.00 |  |
|  | 1-3 times | 1.72 | 1.55 - 1.91 | 2.00 | 1.62 - 2.47 |
|  | 4-5 times | 2.58 | 2.03 - 3.27 | 4.42 | 3.05 - 6.40 |
|  | ≥6 times | 2.12 | 1.70 - 2.63 | 5.35 | 3.94 - 7.26 |
| Feeling lonely | Never | 1.00 |  | 1.00 |  |
|  | Rarely and sometimes | 1.10 | 0.99 - 1.23 | 0.87 | 0.70 - 1.08 |
|  | Often and always | 1.32 | 1.15 - 1.52 | 1.34 | 1.03 - 1.73 |
| Television screen-time | 0 hour | 1.00 |  | 1.00 |  |
|  | < 1 hour | 0.98 | 0.86 - 1.12 | 0.71 | 0.55 - 0.92 |
|  | 1-3 hours | 1.05 | 0.93 - 1.20 | 0.78 | 0.61 - 1.01 |
|  | ≥ 4 hours | 1.33 | 1.12 - 1.58 | 1.92 | 1.44 - 2.56 |
| Video game-time | 0 hour | 1.00 |  | 1.00 |  |
|  | < 1 hour | 0.99 | 0.83 - 1.17 | 0.85 | 0.59 - 1.23 |
|  | 1-3 hours | 1.31 | 1.12 - 1.54 | 1.17 | 0.84 - 1.62 |
|  | ≥ 4 hours | 1.62 | 1.37 - 1.92 | 1.82 | 1.31 - 2.52 |
| Smoking | Never smoking | 1.00 |  | 1.00 |  |
|  | Former smoking | 1.63 | 1.45 - 1.82 | 1.28 | 0.98 - 1.68 |
|  | Current smoking | 5.15 | 4.61 - 5.74 | 7.53 | 6.08 - 9.32 |
| Bullying (A) | No(ref) | 1.00 |  | 1.00 |  |
|  | Yes | 1.04 | 0.94 - 1.15 | 0.78 | 0.64 - 0.96 |
| Bullying (B) | No(ref) | 1.00 |  | 1.00 |  |
|  | Yes | 1.36 | 1.23 - 1.50 | 1.43 | 1.18 - 1.75 |
| Bullying (C) | No(ref) | 1.00 |  | 1.00 |  |
|  | Yes | 0.86 | 0.76 - 0.97 | 0.88 | 0.69 - 1.12 |
| Bullying (D) | No(ref) | 1.00 |  | 1.00 |  |
|  | Yes | 1.03 | 0.90 - 1.18 | 1.33 | 1.02 - 1.73 |
| Bullying (E) | No(ref) | 1.00 |  | 1.00 |  |
|  | Yes | 1.09 | 0.91 - 1.30 | 1.11 | 0.79 - 1.56 |
| Bullying (F) | No(ref) | 1.00 |  | 1.00 |  |
|  | Yes | 1.12 | 0.94 - 1.34 | 1.26 | 0.91 - 1.74 |
| Bullying (G) | No(ref) | 1.00 |  | 1.00 |  |
|  | Yes | 0.96 | 0.79 - 1.17 | 1.47 | 1.06 - 2.06 |

Table S5. Additive effects of **never/former smoking** and bullying victimization on **occasional binge drinking and frequent binge drinking** (N=15,801, model 7)

| **Jointed effects of never/former smoking and bullying victimization on occasional binge drinking and frequent binge drinking** | | | | | | | |
| --- | --- | --- | --- | --- | --- | --- | --- |
| ***Occasional binge drinking*** | | | | | | | |
| Bullying victimization | Smoking | | Bullying victimization & Smoking | | | β | OR（95%CI） |
| No | Never smoking | | 0 | | | — | 1.000 |
| Yes | Never smoking | | 1 | | | 0.227390 | 1.255(1.101 – 1.431) |
| No | Former smoking | | 2 | | | 0.631414 | 1.880(1.575 – 2.245) |
| Yes | Former smoking | | 3 | | | 0.643818 | 1.904(1.631 – 2.223) |
| ***Frequent binge drinking*** | | | | | | | |
| Bullying victimization | Smoking | | Bullying victimization & Smoking | | | β | OR（95%CI） |
| No | Never smoking | | 0 | | | — | 1.000 |
| Yes | Never smoking | | 1 | | | 0.379905 | 1.462(1.054 – 2.028) |
| No | Former smoking | | 2 | | | 0.333540 | 1.396(0.867 – 2.248) |
| Yes | Former smoking | | 3 | | | 0.557629 | 1.747(1.190 – 2.564) |
| **Variance and covariance matrix for never/ former smoking and bullying victimization** | | | | | | | |
| ***Occasional binge drinking*** | | | | | | | |
| Items | | Bullying victimization | | Smoking | Bullying victimization & Smoking | | |
| Bullying victimization | | 0.004449 | | 0.002466 | 0.002777 | | |
| Smoking | | 0.002466 | | 0.008170 | 0.002645 | | |
| Bullying victimization & Smoking | | 0.002777 | | 0.002645 | 0.006245 | | |
| ***Frequent binge drinking*** | | | | | | | |
| Items | | Bullying victimization | | Smoking | Bullying victimization & Smoking | | |
| Bullying victimization | | 0.000545 | | 0.000244 | 0.000281 | | |
| Smoking | | 0.000244 | | 0.001366 | 0.000253 | | |
| Bullying victimization & Smoking | | 0.000281 | | 0.000253 | 0.001049 | | |
| **Results of additive effects for never/former smoking and bullying victimization on occasional binge drinking and frequent binge drinking using Andersson’s Excel** | | | | | | | |
| ***Occasional binge drinking*** | | | | | | | |
| **Exposure** |  | |  | | | OR | 95 CI% |
| Bullying victimization | | | | | | 1.255 | 1.101 – 1.431 |
| Smoking | | | | | | 1.880 | 1.575 – 2.245 |
| Bullying victimization &Smoking | | | | | | 1.904 | 1.631 – 2.223 |
| **Measure** |  | |  | | | Estimate | 95 CI% |
| RERI |  | |  | | | -0.232 | -0.614 – 0.150 |
| AP |  | |  | | | -0.122 | -0.327 – 0.084 |
| S |  | |  | | | 0.796 | 0.557 – 1.137 |
| ***Frequent binge drinking*** | | | | | | | |
| **Exposure** |  | |  | | | OR | 95 CI% |
| Bullying victimization | | | | | | 1.462 | 1.397 – 1.531 |
| Smoking | | | | | | 1.396 | 1.298 – 1.501 |
| Bullying victimization &Smoking | | | | | | 1.747 | 1.639 – 1.861 |
| **Measure** |  | |  | | | Estimate | 95 CI% |
| RERI |  | |  | | | -0.112 | -0.255 – 0.032 |
| AP |  | |  | | | -0.064 | -0.148 – 0.020 |
| S |  | |  | | | 0.870 | 0.729 – 1.038 |

Notes: RERI, the relative excess risk due to interaction; AP, the attributable proportion due to interaction; and S, the synergy index.

Table S6. Additive effects of **never/current smoking** and bullying victimization on **occasional binge drinking and frequent binge drinking** (N=15,109, model 7)

| **Jointed effects of never/current smoking and bullying victimization on occasional binge drinking and frequent binge drinking** | | | | | | | |
| --- | --- | --- | --- | --- | --- | --- | --- |
| ***Occasional binge drinking*** | | | | | | | |
| Bullying victimization | Smoking | | Bullying victimization & Smoking | | | β | OR（95%CI） |
| No | Never smoking | | 0 | | | — | 1.000 |
| Yes | Never smoking | | 1 | | | 0.228060 | 1.256(1.103 – 1.431) |
| No | Current smoking | | 2 | | | 1.670721 | 5.316(4.478 – 6.311) |
| Yes | Current smoking | | 3 | | | 1.821210 | 6.179(5.315 – 7.184) |
| ***Frequent binge drinking*** | | | | | | | |
| Bullying victimization | Smoking | | Bullying victimization & Smoking | | | β | OR（95%CI） |
| No | Never smoking | | 0 | | | — | 1.000 |
| Yes | Never smoking | | 1 | | | 0.337098 | 1.401(1.013 – 1.937) |
| No | Current smoking | | 2 | | | 2.147391 | 8.562(6.054 – 12.111) |
| Yes | Current smoking | | 3 | | | 2.323980 | 10.216(7.418 – 14.069) |
| **Variance and covariance matrix for never/current smoking and bullying victimization** | | | | | | | |
| ***Occasional binge drinking*** | | | | | | | |
| Items | | Bullying victimization | | Smoking | Bullying victimization & Smoking | | |
| Bullying victimization | | 0.004426 | | 0.002559 | 0.002756 | | |
| Smoking | | 0.002559 | | 0.007665 | 0.003125 | | |
| Bullying victimization & Smoking | | 0.002756 | | 0.003125 | 0.005904 | | |
| ***Frequent binge drinking*** | | | | | | | |
| Items | | Bullying victimization | | Smoking | Bullying victimization & Smoking | | |
| Bullying victimization | | 0.000606 | | 0.000241 | 0.000320 | | |
| Smoking | | 0.000241 | | 0.002381 | 0.000322 | | |
| Bullying victimization & Smoking | | 0.000320 | | 0.000322 | 0.001669 | | |
| **Results of additive effects for never/current smoking and bullying victimization on occasional binge drinking and frequent binge drinking using Andersson’s Excel** | | | | | | | |
| ***Occasional binge drinking*** | | | | | | | |
| **Exposure** |  | |  | | | OR | 95 CI% |
| Bullying victimization | | | | | | 1.256 | 1.103 – 1.431 |
| Smoking | | | | | | 5.316 | 4.478 – 6.311 |
| Bullying victimization &Smoking | | | | | | 6.179 | 5.315 – 7.184 |
| **Measure** |  | |  | | | Estimate | 95 CI% |
| RERI |  | |  | | | 0.607 | -0.343 – 1.558 |
| AP |  | |  | | | 0.098 | -0.050 – 0.246 |
| S |  | |  | | | 1.133 | 0.929 -1.384 |
| ***Frequent binge drinking*** | | | | | | | |
| **Exposure** |  | |  | | | OR | 95 CI% |
| Bullying victimization | | | | | | 1.401 | 1.335 – 1.470 |
| Smoking | | | | | | 8.562 | 7.782 – 9.422 |
| Bullying victimization &Smoking | | | | | | 10.216 | 9.430 – 11.068 |
| **Measure** |  | |  | | | Estimate | 95 CI% |
| RERI |  | |  | | | 1.253 | 0.197 – 2.309 |
| AP |  | |  | | | 0.123 | 0.025 – 0.220 |
| S |  | |  | | | 1.157 | 1.022 – 1.310 |

Notes: RERI, the relative excess risk due to interaction; AP, the attributable proportion due to interaction; and S, the synergy index.

Table S7. Multinomial logistic model for smoking×bullying victimization and sociodemographic factors associated with occasional binge drinking and frequent binge drinking among boys, N=10,188 (model 10).

| Variables | | Occasional binge drinking (1-5days) | | Frequent binge drinking (6-30 days) | |
| --- | --- | --- | --- | --- | --- |
|  |  | OR | 95% CI | OR | 95% CI |
| Age |  | 1.06 | 0.99 - 1.12 | 1.15 | 1.03 - 1.28 |
| School type | Middle school (ref) | 1.00 |  | 1.00 |  |
|  | High school | 1.60 | 1.31 - 1.96 | 1.44 | 0.99 - 2.09 |
| Mother`s education level | JHSB | 1.00 |  | 1.00 |  |
|  | STTJ | 1.12 | 0.98 - 1.27 | 1.05 | 0.82 - 1.34 |
|  | Graduate of university and above | 1.11 | 0.94 - 1.30 | 1.45 | 1.07 - 1.95 |
|  | Not sure | 1.07 | 0.85 - 1.35 | 1.23 | 0.84 - 1.81 |
| Boarding students | Yes (ref) | 1.00 |  | 1.00 |  |
|  | No | 1.08 | 0.95 - 1.22 | 1.16 | 0.91 - 1.47 |
| School achievement | Poor (ref) | 1.00 |  | 1.00 |  |
|  | Middle | 1.00 | 0.87 - 1.14 | 0.99 | 0.75 - 1.31 |
|  | Excellent | 0.80 | 0.70 - 0.92 | 1.42 | 1.10 - 1.82 |
|  | Not sure | 1.07 | 0.87 - 1.31 | 1.69 | 1.21 - 2.38 |
| Frequency of fighting | 0 time (ref) | 1.00 |  | 1.00 |  |
|  | 1-3 times | 1.64 | 1.45 - 1.86 | 1.93 | 1.52 - 2.46 |
|  | 4-5 times | 2.86 | 2.20 - 3.73 | 4.83 | 3.21 - 7.26 |
|  | ≥6 times | 2.23 | 1.74 - 2.86 | 5.63 | 4.02 - 7.88 |
| Feeling lonely | Never (ref) | 1.00 |  | 1.00 |  |
|  | Rarely and sometimes | 1.15 | 1.01 - 1.31 | 0.82 | 0.64 - 1.04 |
|  | Often and always | 1.38 | 1.17 - 1.64 | 1.46 | 1.10 - 1.93 |
| Television screen-time | 0 hour (ref) | 1.00 |  | 1.00 |  |
|  | < 1 hour | 0.93 | 0.79 - 1.09 | 0.73 | 0.54 - 0.98 |
|  | 1-3 hours | 0.97 | 0.83 - 1.14 | 0.73 | 0.55 - 0.98 |
|  | ≥ 4 hours | 1.33 | 1.06 - 1.66 | 2.13 | 1.52 - 2.99 |
| Video game-time | 0 hour (ref) | 1.00 |  | 1.00 |  |
|  | < 1 hour | 1.07 | 0.86 - 1.34 | 1.00 | 0.65 - 1.54 [/ |
|  | 1-3 hours | 1.37 | 1.12 - 1.69 | 1.24 | 0.84 - 1.83 / |
|  | ≥ 4 hours | 1.55 | 1.25 - 1.92 | 1.86 | 1.26 - 2.74 ×- |
| **Joint Effects of Smoking and Bullying victimization** | | | | | |
| *Never smoker× No victimization experience* | | 1.00 |  | 1.00 |  |
| *Never smoker× Yes victimization experiences* | | 1.19 | 1.00 - 1.41 | 1.44 | 0.97 - 2.14 |
| *Former smoker× No victimization experience* | | 1.53 | 1.19 - 1.96 | 1.23 | 0.66 - 2.26 |
| *Former smoker × Yes victimization experiences* | | 1.61 | 1.32 - 1.96 | 1.59 | 1.01 - 2.52 |
| *Current smoker× No victimization experience* | | 4.18 | 3.39 - 5.16 | 7.15 | 4.72 - 10.83 |
| *Current smoker× Yes victimization experiences* | | 5.02 | 4.17 - 6.03 | 8.96 | 6.10 - 13.16 |

Table S8. Additive effects of **never/former smoking** and bullying victimization on **occasional binge drinking and frequent binge drinking** among **boys** (N=7,915, model 11)

| **Jointed effects of never/former smoking and bullying victimization on occasional binge drinking and frequent binge drinking** | | | | | | | |
| --- | --- | --- | --- | --- | --- | --- | --- |
| ***Occasional binge drinking*** | | | | | | | |
| Bullying victimization | Smoking | | Bullying victimization & Smoking | | | β | OR（95%CI） |
| No | Never smoking | | 0 | | | — | 1.000 |
| Yes | Never smoking | | 1 | | | 0.182810 | 1.201(1.009 – 1.429) |
| No | Former smoking | | 2 | | | 0.435280 | 1.545(1.205 – 1.981) |
| Yes | Former smoking | | 3 | | | 0.487569 | 1.628(1.330 – 1.994) |
| ***Frequent binge drinking*** | | | | | | | |
| Bullying victimization | Smoking | | Bullying victimization & Smoking | | | β | OR（95%CI） |
| No | Never smoking | | 0 | | | — | 1.000 |
| Yes | Never smoking | | 1 | | | 0.419947 | 1.522(1.019 – 2.273) |
| No | Former smoking | | 2 | | | 0.221578 | 1.248(0.675 – 2.307) |
| Yes | Former smoking | | 3 | | | 0.517546 | 1.678(1.048 – 2.686) |
| **Variance and covariance matrix for never/ former smoking and bullying victimization** | | | | | | | |
| ***Occasional binge drinking*** | | | | | | | |
| Items | | Bullying victimization | | Smoking | Bullying victimization & Smoking | | |
| Bullying victimization | | 0.007875 | | 0.004730 | 0.005151 | | |
| Smoking | | 0.004730 | | 0.016074 | 0.005001 | | |
| Bullying victimization & Smoking | | 0.005151 | | 0.005001 | 0.010683 | | |
| ***Frequent binge drinking*** | | | | | | | |
| Items | | Bullying victimization | | Smoking | Bullying victimization & Smoking | | |
| Bullying victimization | | 0.001078 | | 0.000570 | 0.000628 | | |
| Smoking | | 0.000570 | | 0.002797 | 0.000603 | | |
| Bullying victimization & Smoking | | 0.000628 | | 0.000603 | 0.001858 | | |
| **Results of additive effects for never/former smoking and bullying victimization on occasional binge drinking and frequent binge drinking using Andersson’s Excel** | | | | | | | |
| ***Occasional binge drinking*** | | | | | | | |
| **Exposure** |  | |  | | | OR | 95 CI% |
| Bullying victimization | | | | | | 1.201 | 1.009 – 1.429 |
| Smoking | | | | | | 1.545 | 1.205 – 1.981 |
| Bullying victimization &Smoking | | | | | | 1.628 | 1.330 – 1.994 |
| **Measure** |  | |  | | | Estimate | 95 CI% |
| RERI |  | |  | | | -0.118 | -0.557 – 0.322 |
| AP |  | |  | | | -0.072 | -0.345 – 0.200 |
| S |  | |  | | | 0.842 | 0.459 – 1.546 |
| ***Frequent binge drinking*** | | | | | | | |
| **Exposure** |  | |  | | | OR | 95 CI% |
| Bullying victimization | | | | | | 1.522 | 1.427 – 1.623 |
| Smoking | | | | | | 1.248 | 1.125 – 1.384 |
| Bullying victimization &Smoking | | | | | | 1.678 | 1.542 – 1.826 |
| **Measure** |  | |  | | | Estimate | 95 CI% |
| RERI |  | |  | | | -0.092 | -0.273 – 0.089 |
| AP |  | |  | | | -0.055 | -0.164 – 0.055 |
| S |  | |  | | | 0.880 | 0.689 – 1.125 |

Notes: RERI, the relative excess risk due to interaction; AP, the attributable proportion due to interaction; and S, the synergy index.

Table S9. Additive effects of **never/current smoking** and bullying victimization on **occasional binge drinking and frequent binge drinking** among **boys** (N=8,119, model 11)

| **Jointed effects of never/current smoking and bullying victimization on occasional binge drinking and frequent binge drinking** | | | | | | | |
| --- | --- | --- | --- | --- | --- | --- | --- |
| ***Occasional binge drinking*** | | | | | | | |
| Bullying victimization | Smoking | | Bullying victimization & Smoking | | | β | OR（95%CI） |
| No | Never smoking | | 0 | | | — | 1.000 |
| Yes | Never smoking | | 1 | | | 0.146866 | 1.158(0.974 – 1.377) |
| No | Current smoking | | 2 | | | 1.408694 | 4.091(3.312 – 5.052) |
| Yes | Current smoking | | 3 | | | 1.577748 | 4.844(4.018 – 5.840) |
| ***Frequent binge drinking*** | | | | | | | |
| Bullying victimization | Smoking | | Bullying victimization & Smoking | | | β | OR（95%CI） |
| No | Never smoking | | 0 | | | — | 1.000 |
| Yes | Never smoking | | 1 | | | 0.335356 | 1.398(0.940 – 2.081) |
| No | Current smoking | | 2 | | | 1.964588 | 7.132(4.697 – 10.829) |
| Yes | Current smoking | | 3 | | | 2.176352 | 8.814(5.979 – 12.994) |
| **Variance and covariance matrix for never/current smoking and bullying victimization** | | | | | | | |
| ***Occasional binge drinking*** | | | | | | | |
| Items | | Bullying victimization | | Smoking | Bullying victimization & Smoking | | |
| Bullying victimization | | 0.007786 | | 0.004689 | 0.004972 | | |
| Smoking | | 0.004689 | | 0.011598 | 0.005450 | | |
| Bullying victimization & Smoking | | 0.004972 | | 0.005450 | 0.009109 | | |
| ***Frequent binge drinking*** | | | | | | | |
| Items | | Bullying victimization | | Smoking | Bullying victimization & Smoking | | |
| Bullying victimization | | 0.001161 | | 0.000538 | 0.000651 | | |
| Smoking | | 0.000538 | | 0.003397 | 0.000703 | | |
| Bullying victimization & Smoking | | 0.000651 | | 0.000703 | 0.002435 | | |
| **Results of additive effects for never/current smoking and bullying victimization on occasional binge drinking and frequent binge drinking using Andersson’s Excel** | | | | | | | |
| ***Occasional binge drinking*** | | | | | | | |
| **Exposure** |  | |  | | | OR | 95 CI% |
| Bullying victimization | | | | | | 1.158 | 0.974 – 1.377 |
| Smoking | | | | | | 4.091 | 3.312 – 5.052 |
| Bullying victimization &Smoking | | | | | | 4.844 | 4.018 – 5.840 |
| **Measure** |  | |  | | | Estimate | 95 CI% |
| RERI |  | |  | | | 0.595 | -0.261 – 1.452 |
| AP |  | |  | | | 0.123 | -0.046 – 0.292 |
| S |  | |  | | | 1.183 | 0.921 – 1.520 |
| ***Frequent binge drinking*** | | | | | | | |
| **Exposure** |  | |  | | | OR | 95 CI% |
| Bullying victimization | | | | | | 1.398 | 1.308 – 1.495 |
| Smoking | | | | | | 7.132 | 6.362 – 7.995 |
| Bullying victimization &Smoking | | | | | | 8.814 | 8.002 – 9.709 |
| **Measure** |  | |  | | | Estimate | 95 CI% |
| RERI |  | |  | | | 1.284 | 0.264 – 2.303 |
| AP |  | |  | | | 0.146 | 0.038 – 0.253 |
| S |  | |  | | | 1.197 | 1.036 – 1.382 |

Notes: RERI, the relative excess risk due to interaction; AP, the attributable proportion due to interaction; and S, the synergy index.

Table S10. Multinomial logistic model for smoking×bullying victimization and sociodemographic factors associated with occasional binge drinking and frequent binge drinking among girls, N=8,587 (model 8).

| Variables | | Occasional binge drinking (1-5days) | | Frequent binge drinking (6-30 days) | |
| --- | --- | --- | --- | --- | --- |
|  |  | OR | 95% CI | OR | 95% CI |
| Age |  | 1.01 | 0.94 - 1.10 | 1.24 | 1.03 - 1.50 |
| School type | Middle school (ref) | 1.00 |  | 1.00 |  |
|  | High school | 0.93 | 0.71 - 1.21 | 0.97 | 0.50 - 1.87 |
| Mother`s education level | JHSB | 1.00 |  | 1.00 |  |
|  | STTJ | 1.23 | 1.05 - 1.45 | 1.30 | 0.88 - 1.93 |
|  | Graduate of university and above | 1.23 | 0.98 - 1.53 | 0.63 | 0.32 - 1.24 |
|  | Not sure | 0.91 | 0.63 - 1.32 | 1.64 | 0.82 - 3.29 |
| Boarding students | Yes (ref) | 1.00 |  | 1.00 |  |
|  | No | 1.00 | 0.85 - 1.18 | 1.45 | 0.97 - 2.18 |
| School achievement | Poor (ref) | 1.00 |  |  |  |
|  | Middle | 0.94 | 0.78 - 1.13 | 0.97 | 0.63 - 1.48 |
|  | Excellent | 0.92 | 0.76 - 1.10 | 0.78 | 0.49 - 1.23 |
|  | Not sure | 1.15 | 0.84 - 1.57 | 1.16 | 0.60 - 2.24 |
| Frequency of fighting | 0 time (ref) | 1.00 |  | 1.00 |  |
|  | 1-3 times | 2.09 | 1.73 - 2.52 | 2.43 | 1.58 - 3.74 |
|  | 4-5 times | 2.39 | 1.39 - 4.10 | 5.69 | 2.39 - 13.56 |
|  | ≥6 times | 2.87 | 1.82 - 4.52 | 10.38 | 5.34 - 20.18 |
| Feeling lonely | Never (ref) | 1.00 |  | 1.00 |  |
|  | Rarely and sometimes | 1.01 | 0.84 - 1.22 | 1.16 | 0.70 - 1.91 |
|  | Often and always | 1.17 | 0.93 - 1.47 | 1.62 | 0.93 - 2.82 |
| Television screen-time | 0 hour (ref) | 1.00 |  | 1.00 |  |
|  | < 1 hour | 1.09 | 0.86 - 1.38 | 0.57 | 0.33 - 0.99 |
|  | 1-3 hours | 1.25 | 1.00 - 1.58 | 0.82 | 0.50 - 1.34 |
|  | ≥ 4 hours | 1.36 | 1.02 - 1.81 | 1.44 | 0.82 - 2.51 |
| Video game-time | 0 hour (ref) | 1.00 |  | 1.00 |  |
|  | < 1 hour | 0.92 | 0.69 - 1.21 | 0.55 | 0.27 - 1.09 |
|  | 1-3 hours | 1.27 | 0.98 - 1.65 | 0.85 | 0.46 - 1.56 |
|  | ≥ 4 hours | 1.92 | 1.45 - 2.54 | 1.52 | 0.82 - 2.82 |
| **Joint Effects of Smoking and Bullying victimization** | | | | | |
| *Never smoker× No victimization experience* | | 1.00 |  | 1.00 |  |
| *Never smoker× Yes victimization experiences* | | 1.33 | 1.09 - 1.62 | 1.25 | 0.71 - 2.22 |
| *Former smoker× No victimization experience* | | 2.37 | 1.84 - 3.05 | 1.65 | 0.78 - 3.50 |
| *Former smoker × Yes victimization experiences* | | 2.36 | 1.86 - 3.00 | 1.84 | 0.96 - 3.54 |
| *Current smoker× No victimization experience* | | 8.17 | 6.04 - 11.07 | 11.71 | 6.21 - 22.06 |
| *Current smoker× Yes victimization experiences* | | 9.17 | 7.07 - 11.89 | 12.37 | 7.01 - 21.85 |

Table S11. Additive effects of **never/former smoking** and bullying victimization on **occasional binge drinking and frequent binge drinking** among **girls** (N=7,886, model 9)

| **Jointed effects of never/former smoking and bullying victimization on occasional binge drinking and frequent binge drinking** | | | | | | | |
| --- | --- | --- | --- | --- | --- | --- | --- |
| ***Occasional binge drinking*** | | | | | | | |
| Bullying victimization | Smoking | | Bullying victimization & Smoking | | | β | OR（95%CI） |
| No | Never smoking | | 0 | | | — | 1.000 |
| Yes | Never smoking | | 1 | | | 0.291218 | 1.338(1.100 – 1.627) |
| No | Former smoking | | 2 | | | 0.872612 | 2.393(1.854 – 3.089) |
| Yes | Former smoking | | 3 | | | 0.870160 | 2.387(1.881 – 3.030) |
| ***Frequent binge drinking*** | | | | | | | |
| Bullying victimization | Smoking | | Bullying victimization & Smoking | | | β | OR（95%CI） |
| No | Never smoking | | 0 | | | — | 1.000 |
| Yes | Never smoking | | 1 | | | 0.247698 | 1.281(0.724 – 2.266) |
| No | Former smoking | | 2 | | | 0.482455 | 1.620(0.757 – 3.465) |
| Yes | Former smoking | | 3 | | | 0.631581 | 1.881(0.978 – 3.617) |
| **Variance and covariance matrix for never/ former smoking and bullying victimization** | | | | | | | |
| ***Occasional binge drinking*** | | | | | | | |
| Items | | Bullying victimization | | Smoking | Bullying victimization & Smoking | | |
| Bullying victimization | | 0.009957 | | 0.005029 | 0.005390 | | |
| Smoking | | 0.005029 | | 0.016950 | 0.005515 | | |
| Bullying victimization & Smoking | | 0.005390 | | 0.005515 | 0.014797 | | |
| ***Frequent binge drinking*** | | | | | | | |
| Items | | Bullying victimization | | Smoking | Bullying victimization & Smoking | | |
| Bullying victimization | | 0.001052 | | 0.000382 | 0.000386 | | |
| Smoking | | 0.000382 | | 0.002669 | 0.000375 | | |
| Bullying victimization & Smoking | | 0.000386 | | 0.000375 | 0.002415 | | |
| **Results of additive effects for never/former smoking and bullying victimization on occasional binge drinking and frequent binge drinking using Andersson’s Excel** | | | | | | | |
| ***Occasional binge drinking*** | | | | | | | |
| **Exposure** |  | |  | | | OR | 95 CI% |
| Bullying victimization | | | | | | 1.338 | 1.100 - 1.627 |
| Smoking | | | | | | 2.393 | 1.854 - 3.089 |
| Bullying victimization &Smoking | | | | | | 2.387 | 1.881 – 3.030 |
| **Measure** |  | |  | | | Estimate | 95 CI% |
| RERI |  | |  | | | -0.344 | -1.061 - 0.374 |
| AP |  | |  | | | -0.144 | -0.458 - 0.170 |
| S |  | |  | | | 0.801 | 0.512 – 1.255 |
| ***Frequent binge drinking*** | | | | | | | |
| **Exposure** |  | |  | | | OR | 95 CI% |
| Bullying victimization | | | | | | 1.281 | 1.202 – 1.356 |
| Smoking | | | | | | 1.620 | 1.464 – 1.793 |
| Bullying victimization &Smoking | | | | | | 1.881 | 1.708 – 2.071 |
| **Measure** |  | |  | | | Estimate | 95 CI% |
| RERI |  | |  | | | -0.021 | -0.258 – 0.217 |
| AP |  | |  | | | -0.011 | -0.138 – 0.116 |
| S |  | |  | | | 0.977 | 0.749 – 1.276 |

Notes: RERI, the relative excess risk due to interaction; AP, the attributable proportion due to interaction; and S, the synergy index.

Table S12. Additive effects of **never/current smoking** and bullying victimization on **occasional binge drinking and frequent binge drinking** among **girls** (N=6,990, model 9)

| **Jointed effects of never/current smoking and bullying victimization on occasional binge drinking and frequent binge drinking** | | | | | | | |
| --- | --- | --- | --- | --- | --- | --- | --- |
| ***Occasional binge drinking*** | | | | | | | |
| Bullying victimization | Smoking | | Bullying victimization & Smoking | | | β | OR（95%CI） |
| No | Never smoking | | 0 | | | — | 1.000 |
| Yes | Never smoking | | 1 | | | 0.276718 | 1.319(1.080 – 1.611) |
| No | Current smoking | | 2 | | | 2.068282 | 7.911(5.824 – 10.747) |
| Yes | Current smoking | | 3 | | | 2.181290 | 8.858(6.795 – 11.547) |
| ***Frequent binge drinking*** | | | | | | | |
| Bullying victimization | Smoking | | Bullying victimization & Smoking | | | β | OR（95%CI） |
| No | Never smoking | | 0 | | | — | 1.000 |
| Yes | Never smoking | | 1 | | | 0.213667 | 1.238(0.696 – 2.202) |
| No | Current smoking | | 2 | | | 2.497225 | 12.149(6.416 – 23.004) |
| Yes | Current smoking | | 3 | | | 2.528989 | 12.541(7.020 – 22.403) |
| **Variance and covariance matrix for never/current smoking and bullying victimization** | | | | | | | |
| ***Occasional binge drinking*** | | | | | | | |
| Items | | Bullying victimization | | Smoking | Bullying victimization & Smoking | | |
| Bullying victimization | | 0.010430 | | 0.005302 | 0.005956 | | |
| Smoking | | 0.005302 | | 0.024430 | 0.006469 | | |
| Bullying victimization & Smoking | | 0.005956 | | 0.006469 | 0.018304 | | |
| ***Frequent binge drinking*** | | | | | | | |
| Items | | Bullying victimization | | Smoking | Bullying victimization & Smoking | | |
| Bullying victimization | | 0.001252 | | 0.000340 | 0.000513 | | |
| Smoking | | 0.000340 | | 0.008560 | 0.000252 | | |
| Bullying victimization & Smoking | | 0.000513 | | 0.000252 | 0.006103 | | |
| **Results of additive effects for never/current smoking and bullying victimization on occasional binge drinking and frequent binge drinking using Andersson’s Excel** | | | | | | | |
| ***Occasional binge drinking*** | | | | | | | |
| **Exposure** |  | |  | | | OR | 95 CI% |
| Bullying victimization | | | | | | 1.319 | 1.080 – 1.611 |
| Smoking | | | | | | 7.911 | 5.824 – 10.747 |
| Bullying victimization &Smoking | | | | | | 8.858 | 6.795 – 11.547 |
| **Measure** |  | |  | | | Estimate | 95 CI% |
| RERI |  | |  | | | 0.628 | -2.177 – 3.433 |
| AP |  | |  | | | 0.071 | -0.236 – 0.378 |
| S |  | |  | | | 1.087 | 0.747 – 1.581 |
| ***Frequent binge drinking*** | | | | | | | |
| **Exposure** |  | |  | | | OR | 95 CI% |
| Bullying victimization | | | | | | 1.238 | 1.155 – 1.327 |
| Smoking | | | | | | 12.149 | 10.134 – 14.564 |
| Bullying victimization &Smoking | | | | | | 12.541 | 10.760 – 14.616 |
| **Measure** |  | |  | | | Estimate | 95 CI% |
| RERI |  | |  | | | 0.154 | -2.715 – 3.023 |
| AP |  | |  | | | 0.012 | -0.215 – 0.240 |
| S |  | |  | | | 1.014 | 0.789 – 1.302 |

Notes: RERI, the relative excess risk due to interaction; AP, the attributable proportion due to interaction; and S, the synergy index.

Table S13. Multivariate logistic model for factors associated with ever drinking, N=31,825 (model 1, full results).

| Variables | | Outcome is drinker/non-drinker | |
| --- | --- | --- | --- |
|  |  | OR | 95%CI |
| Gender | Boys (ref) | 1.00 |  |
|  | Girls | 0.86 | 0.82 - 0.91 |
| Age |  | 1.08 | 1.06 - 1.11 |
| School type | Middle school (ref) | 1.00 |  |
|  | High school | 1.59 | 1.46 - 1.74 |
| Mother`s education level | JHSB (ref) | 1.00 |  |
|  | STTJ | 1.16 | 1.09 - 1.23 |
|  | Graduate of university and above | 1.24 | 1.15 - 1.33 |
|  | Not sure | 0.89 | 0.80 - 1.00 |
| Boarding students | Yes (ref) | 1.00 |  |
|  | No | 1.13 | 1.06 - 1.21 |
| School achievement | Poor (ref) | 1.00 |  |
|  | Middle | 0.87 | 0.81 - 0.93 |
|  | Excellent | 0.81 | 0.76 - 0.87 |
|  | Not sure | 0.76 | 0.68 - 0.85 |
| Frequency of fighting, past 12 months | 0 time (ref) | 1.00 |  |
|  | 1-3 times | 1.61 | 1.49 - 1.73 |
|  | 4-5 times | 1.71 | 1.37 - 2.13 |
|  | ≥ 6 times | 2.06 | 1.68 - 2.52 |
| Feeling lonely | Never (ref) | 1.00 |  |
|  | Rarely or sometimes | 1.49 | 1.41 - 1.57 |
|  | Often or always | 1.83 | 1.68 - 2.00 |
| Television screen-time | 0 hour (ref) | 1.00 |  |
|  | < 1 hour | 1.06 | 0.98 - 1.14 |
|  | 1-3 hours | 1.14 | 1.05 - 1.22 |
|  | ≥ 4 hours | 1.23 | 1.09 - 1.38 |
| Video game-time | 0 hour (ref) | 1.00 |  |
|  | < 1 hour | 1.25 | 1.16 - 1.36 |
|  | 1-3 hours | 1.67 | 1.54 - 1.80 |
|  | ≥ 4 hours | 2.19 | 1.98 - 2.42 |
| Smoking | Never (ref) | 1.00 |  |
|  | Former | 4.28 | 3.91 - 4.68 |
|  | Current | 4.63 | 4.13 - 5.20 |
| Bullying (A) | No (ref) | 1.00 |  |
|  | Yes | 1.11 | 1.05 - 1.18 |
| Bullying (B) | No (ref) | 1.00 |  |
|  | Yes | 1.60 | 1.49 - 1.72 |
| Bullying (C) | No (ref) | 1.00 |  |
|  | Yes | 1.12 | 1.03 - 1.21 |
| Bullying (D) | No (ref) | 1.00 |  |
|  | Yes | 0.81 | 0.74 - 0.88 |
| Bullying (E) | No (ref) | 1.00 |  |
|  | Yes | 0.92 | 0.81 - 1.04 |
| Bullying (F) | No (ref) | 1.00 |  |
|  | Yes | 1.01 | 0.89 - 1.14 |
| Bullying (G) | No (ref) | 1.00 |  |
|  | Yes | 0.73 | 0.64 - 0.84 |

Notes: (A) Maliciously teased (B) Made fun of with gender jokes, comments or gestures (C) Been made fun of due to individual physical imperfections or looks (D) Deliberately excluded from or outside of a collective activity (E) Threatened or intimidated (F) Blackmailed or forced to give money (G) Been hit, kicked, pushed, squeezed, or locked indoors. Model 1: The logistic regression model for smoking and different type of bullying victimization affecting drinking status adjusting for sociodemographic factors among all participants.

Table S14. Multinomial logistic model for smoking and bullying victimization interacted in their association with binge drinking among ever drinkers by adjustment for sociodemographic factors.

| Variables | | Model 2, N=18,775 | | Model 3, N=18,775 | | | |
| --- | --- | --- | --- | --- | --- | --- | --- |
|  |  | Outcome is none/binge drinking (1- 30 days)^a^ | | Occasional binge drinking (1-5days)^b^ | | Frequent binge drinking (6-30 days)^c^ | |
|  |  | OR | 95% CI | OR | 95% CI | OR | 95% CI |
| Gender | Boys (ref) | 1.00 |  | 1.00 |  | 1.00 |  |
|  | Girls | 0.82 | 0.75 - 0.90 | 0.85 | 0.78 - 0.94 | 0.65 | 0.53 - 0.80 |
| Age |  | 1.05 | 1.01 - 1.10 | 1.03 | 0.99 - 1.08 | 1.16 | 1.06 - 1.28 |
| School type | Middle school (ref) | 1.00 |  | 1.00 |  | 1.00 |  |
|  | High school | 1.30 | 1.12 - 1.52 | 1.30 | 1.11 - 1.53 | 1.28 | 0.93 - 1.77 |
| Mother`s education level | JHSB | 1.00 |  | 1.00 |  | 1.00 |  |
|  | STTJ | 1.17 | 1.06 - 1.29 | 1.17 | 1.06 - 1.30 | 1.13 | 0.92 - 1.39 |
|  | Graduate of university and above | 1.19 | 1.05 - 1.35 | 1.17 | 1.02 - 1.33 | 1.29 | 0.99 - 1.69 |
|  | Not sure | 1.09 | 0.91 - 1.31 | 1.04 | 0.86 - 1.26 | 1.32 | 0.94 - 1.84 |
| Boarding students | Yes (ref) | 1.00 |  | 1.00 |  | 1.00 |  |
|  | No | 1.09 | 0.99 - 1.20 | 1.06 | 0.96 - 1.17 | 1.23 | 1.00 - 1.50 |
| School achievement | Poor (ref) | 1.00 |  | 1.00 |  | 1.00 |  |
|  | Middle | 0.98 | 0.89 - 1.09 | 0.98 | 0.88 - 1.09 | 1.00 | 0.79 - 1.26 |
|  | Excellent | 0.90 | 0.81 - 1.00 | 0.85 | 0.76 - 0.95 | 1.25 | 1.00 - 1.56 |
|  | Not sure | 1.16 | 0.99 - 1.36 | 1.09 | 0.92 - 1.30 | 1.55 | 1.15 - 2.10 |
| Frequency of fighting | 0 time (ref) | 1.00 |  | 1.00 |  | 1.00 |  |
|  | 1-3 times | 1.79 | 1.62 - 1.98 | 1.75 | 1.58 - 1.94 | 2.08 | 1.69 - 2.57 |
|  | 4-5 times | 2.97 | 2.38 - 3.70 | 2.65 | 2.10 - 3.36 | 4.88 | 3.38 - 7.04 |
|  | ≥6 times | 2.85 | 2.35 - 3.46 | 2.23 | 1.79 - 2.77 | 6.21 | 4.61 - 8.37 |
| Feeling lonely | Never (ref) | 1.00 |  | 1.00 |  | 1.00 |  |
|  | Rarely and sometimes | 1.07 | 0.96 - 1.18 | 1.10 | 0.99 - 1.23 | 0.87 | 0.70 - 1.08 |
|  | Often and always | 1.36 | 1.20 - 1.54 | 1.33 | 1.16 - 1.52 | 1.46 | 1.14 - 1.87 |
| Television screen-time | 0 hour (ref) | 1.00 |  | 1.00 |  | 1.00 |  |
|  | < 1 hour | 0.92 | 0.81 - 1.04 | 0.97 | 0.85 - 1.11 | 0.68 | 0.53 - 0.89 |
|  | 1-3 hours | 0.99 | 0.88 - 1.12 | 1.04 | 0.92 - 1.19 | 0.75 | 0.58 - 0.96 |
|  | ≥ 4 hours | 1.43 | 1.22 - 1.68 | 1.32 | 1.11 - 1.57 | 1.92 | 1.44 - 2.56 |
| Video game-time | 0 hour (ref) | 1.00 |  | 1.00 |  | 1.00 |  |
|  | < 1 hour | 0.96 | 0.82 - 1.13 | 0.99 | 0.83 - 1.17 | 0.85 | 0.59 - 1.22 |
|  | 1-3 hours | 1.28 | 1.10 - 1.49 | 1.32 | 1.12 - 1.54 | 1.12 | 0.80 - 1.55 |
|  | ≥ 4 hours | 1.66 | 1.41 - 1.94 | 1.64 | 1.38 - 1.94 | 1.77 | 1.27 - 2.45 |
| **Joint Effects of Smoking and Bullying victimization** | | | |  |  |  |  |
| *Never smoker × No victimization experience* |  | 1.00 |  | 1.00 |  | 1.00 |  |
| *Never smoker × Yes victimization experiences* |  | 1.30 | 1.15 - 1.47 | 1.29 | 1.13 - 1.47 | 1.44 | 1.05 - 1.99 |
| *Former smoker × No victimization experience* |  | 1.82 | 1.54 - 2.16 | 1.89 | 1.59 - 2.26 | 1.41 | 0.88 - 2.27 |
| *Former smoker × Yes victimization experiences* |  | 1.91 | 1.66 - 2.21 | 1.96 | 1.68 - 2.28 | 1.76 | 1.21 - 2.55 |
| *Current smoker × No victimization experience* |  | 5.93 | 5.06 - 6.96 | 5.53 | 4.66 - 6.55 | 8.57 | 6.08 - 12.08 |
| *Current smoker × Yes victimization experiences* |  | 7.01 | 6.10 - 8.05 | 6.49 | 5.60 - 7.52 | 10.32 | 7.52 - 14.14 |
